# Identification of hematein as a novel inhibitor of protein kinase CK2 from a natural product library

**DOI:** 10.1186/1471-2407-9-135

**Published:** 2009-05-06

**Authors:** Ming-Szu Hung, Zhidong Xu, Yu-Ching Lin, Jian-Hua Mao, Cheng-Ta Yang, Pey-Jium Chang, David M Jablons, Liang You

**Affiliations:** 1 Thoracic Oncology Laboratory, Department of Surgery, Comprehensive Cancer Center, University of California, San Francisco, CA 94115, USA; 2 Division of Pulmonary and Critical Care Medicine, Chang Gung Memorial Hospital, Chiayi, Taiwan, R.O.C.; 3 Graduate Institute of Clinical Medical Sciences, College of Medicine, Chang Gung University, Taoyuan, Taiwan, R.O.C.; 4 Life Sciences Division, Lawrence Berkeley National Laboratory, University of California, Berkeley, CA, USA; 5 Department of Respiratory Care, College of Medicine, Chang Gung University, Taoyuan, Taiwan, R.O.C.

## Abstract

**Background:**

Casein kinase 2 (CK2) is dysregulated in various human cancers and is a promising target for cancer therapy. To date, there is no small molecular CK2 inhibitor in clinical trial yet. With the aim to identify novel CK2 inhibitors, we screened a natural product library.

**Methods:**

We adopted cell-based proliferation and CK2 kinase assays to screen CK2 inhibitors from a natural compound library. Dose-dependent response of CK2 inhibitors *in vitro *was determined by a radioisotope kinase assay. Western blot analysis was used to evaluate down stream Akt phosphorylation and apoptosis. Apoptosis was also evaluated by annexin-V/propidium iodide (PI) labeling method using flow cytometry. Inhibition effects of CK2 inhibitors on the growth of cancer and normal cells were evaluated by cell proliferation and viability assays.

**Results:**

Hematein was identified as a novel CK2 inhibitor that is highly selective among a panel of kinases. It appears to be an ATP non-competitive and partially reversible CK2 inhibitor with an IC_50 _value of 0.55 μM. In addition, hematein inhibited cancer cell growth partially through down-regulation of Akt phosphorylation and induced apoptosis in these cells. Furthermore, hematein exerted stronger inhibition effects on the growth of cancer cells than in normal cells.

**Conclusion:**

In this study, we showed that hematein is a novel selective and cell permeable small molecule CK2 inhibitor. Hematein showed stronger growth inhibition effects to cancer cells when compared to normal cells. This compound may represent a promising class of CK2 inhibitors.

## Background

CK2 is a serine/threonine protein kinase composed of 2 catalytic subunits (αα, α'α' or αα') and 2 regulatory subunits (β). CK2 is ubiquitously expressed and highly conserved in cells and plays multiple roles in cellular processes, including gene expression, protein synthesis, cell proliferation and apoptosis[1]. So far, CK2 is known to phosphorylate more than 300 proteins in cells and is also an important regulator of intracellular signalling pathways[2]. For example, CK2 promotes survival by increasing survivin expression via beta-catenin-Tcf/Lef-mediated transcription[3]. CK2 also constitutively phosphorylates and upregulates Akt/PKB Ser129 *in vitro *and *in vivo*, which may be required for maximal activation of Akt/PKB[4].

Dysregulation of CK2 in association with other proteins also increases oncogenic potential of cells[5]. In transgenic mouse study, expression of CK2α subunits in lymphocyte induces lymphoma, and the coexpression of c-myc protein results in neonatal leukemia[6]. Overexpression of CK2α in the mammary gland of transgenic mouse induces mammary hyperplasia, dysplasia, and eventually adenocarcinomas[7]. In primary embryo fibroblasts, coexpression of CK2α' and H-Ras induces transformation[8]. Overexpression of CK2 has been noted in a variety of human cancers, including acute myeloid leukaemia[9], mammary gland[7], prostate[10], lung[11], head and neck[12], and kidney cancer[13], and also correlates with metastatic potential, undifferentiated histological type and poor clinical outcome in human cancers. As a result, CK2 is a potential candidate of targeted therapy for cancers[1]. Although CK2 inhibitors like TBB (4,5,6,7 tetrabrome benzotriazole)[14] and its derivatives[1,15] have been shown to induce apoptosis in human cancer cells, more selective CK2 inhibitors are needed, since to our knowledge there is still not one CK2 small molecule inhibitor in clinical trials for cancer treatment yet.

In this study, we screened for potential CK2 inhibitors from a natural compound library via cell based proliferation and kinase assays. Through these assays, hematein was identified as a novel CK2 inhibitor. We further evaluated the dose dependent inhibition response of hematein on CK2 kinase activity *in vitro *and in cancer cells. Effects of hematein on apoptosis and cell growth were also evaluated in cancer and normal cells.

## Methods

### Cell culture

HeLa (CCL-2), HCT116 (CCL-247), A549 (CCL-185), A427 (HTB-53), WI-38 (CCL-75) and CCL-211 cell lines were purchased from American Type Culture Collection (Manassas, VA). Cells were grown in complete growth medium (Dulbecco's modified Eagle's medium for HeLa, A549 and CCL-211; Eagle's Minimum Essential Medium for WI-38; Roswell Park Memorial Institute's medium for HCT116 and A427) supplemented with 10% fetal bovine serum, 10 units/ml penicillin and 10 μg/ml streptomycin at 37°C and 5% CO2.

### Compound library

A natural product library NPL 400 (Timtect Inc., Newark, DE) was used to screen possible CK2 inhibitors. This library is composed of 400 highly pure, rationally selected drug-like small-molecule compounds with molecular weights ranging from 183 to 832 Da. All compounds were 0.5 mg powder form in individual wells of 96-well plates. After dissolved with 100 μL of dimethyl sulfoxide (DMSO), compounds were stored at -20°C with final concentrations of 5 mg/ml. TBB was purchased from Sigma-Aldrich Co (St. Louis, MI).

### Cell proliferation and viability assay

The CellTiter 96^® ^AQ_ueous _One Solution Cell Proliferation Assay (MTS) (Promega, Madison, WI) was used to evaluate growth of normal and cancer cells after treatment by different compounds. Exponentially growing cells were plated in 96-well microtiter plates at 5 × 10^3 ^cells/well with indicated concentrations of compounds. After incubation with indicated amount of compounds for 48 hours, 20 μl of the CellTiter 96^® ^AQ_ueous _One Solution Reagent was added directly to culture wells. Absorbance at 490 nm was recorded with a 96-well plate reader after 2 hours incubation.

CellTiter-Glo luminescent cell viability assay (Promega, Madison, WI) was used to evaluate the cytotoxicity of hematein. After incubation with indicated amount of compounds for 48 hours, 100 μl of the CellTiter-Glo reagent was added directly to culture wells. Following procedures recommended by the manufacture, the luminescence produced by the luciferase-catalyzed reaction of luciferin and ATP was measured using a laminator.

### Kinase assays

The CK2 Kinase Assay/Inhibitor Screening Kit (CycLex Co. Ltd., Japan) was used for further screening of compounds for their CK2 inhibition effects *in vitro *according to manufacture's manual[16].

For determination of the dose dependent inhibition response of hematein, Casein Kinase 2 Assay Kit (Millipore, Bedford, MA) was used according to manufacture's protocol. Kinase assay was carried out in the presence of increasing amount of hematein in a final volume of 50 μl containing 20 mM MOPS (pH 7.2), 25 mM β-glycerol phosphate, 5 mM EGTA, 1 mM sodium orthovanadate, 1 mM dithiothreitol, 15 mM Mgcl_2_, 200 μM for CK2 substrate peptide: RRRDDDSDDD (Millipore, Bedford, MA), 0.05 μg purified active CK2 (Millipore, Bedford, MA) and [γ-33P]-ATP. After incubation in 30°C for 20 minutes, assay was stopped by adding of 20 μl 4% trichloroacetic acid and transferred 25 μl to P81 phosphocellulose squares. After washing with 0.75% phosphoric acid for 6 times and with acetone for 1 time, phosphocellulose squares were dried and transferred to scintillation vials for counting.

### Western blot analysis

After treated with indicated concentration of hematein for 48 hours, whole cell protein were extracted from A549 cells with M-PER Mammalian Protein Extraction Reagent (Pierce, Rockfold, IL) added with Phosphatase Inhibitor Cocktail Set II (Calbiochem, San Diego, CA) and Complete Protease Inhibitor Cocktails (Roche, Switzerland) according to manufactures' protocols. The proteins were used for further CK2 kinase activity assay or western blot analysis. For western blot analysis, the proteins were separated on 4–15% gradient sodium dodecyl sulfate (SDS)-polyacrylamide gels and transferred to Immobilon-P membranes (Millipore, Billerica, MA). Following primary antibodies: Akt, PARP (Cell Signaling Technology, Danvers, MA), phospho-Akt S129 (Abcam Inc., Cambridge, MA) and β-actin (Sigma, St. Louis, MO) were used. After binding to indicated secondary antibodies, an enhanced chemiluminescence (ECL) blotting analysis system (GE Healthcare Life Sciences, Piscataway, NJ) was used for antigen-antibody detection.

### Apoptosis assay

The occurrence of apoptosis was determined by ApoTarget™ annexin V-FITC kit (BioSource International, Inc., Camarillo, CA). Briefly, cells were treated with indicated amounts of DMSO or hematein for 48 hours. The cells were washed with cold PBS and then re-suspended in binding buffer. The cells were aliquot to 100 μl in a concentration of 1 × 10^6^/ml. After adding 5 μl of annexin V-FITC and 20 μl of PI and incubated in dark for 15 minutes, the cells were resuspended in 400 μl of binding buffer. Accuri's C6 Flow Cytometer™ System (Accuri Cytometers, Ann Arbor, MI) was used for analysis.

### Statistical analysis

The data shown represent mean values ± standard error of deviation (SD). Student's *t*-test was used for comparing of cell viability in different treatments. Statistical analysis was carried out using SPSS (version 10.0, Chicago, IL). Significance was defined as p < 0.05 with two sided analysis. The half maximal inhibitory concentration (IC50) values was determined using GraphPad Prism^® ^log (inhibitor) vs. response (variable slope) software (version 5, La Jolla, CA).

## Results

### Screening CK2 inhibitors from a natural compound library

**Figure 1:**
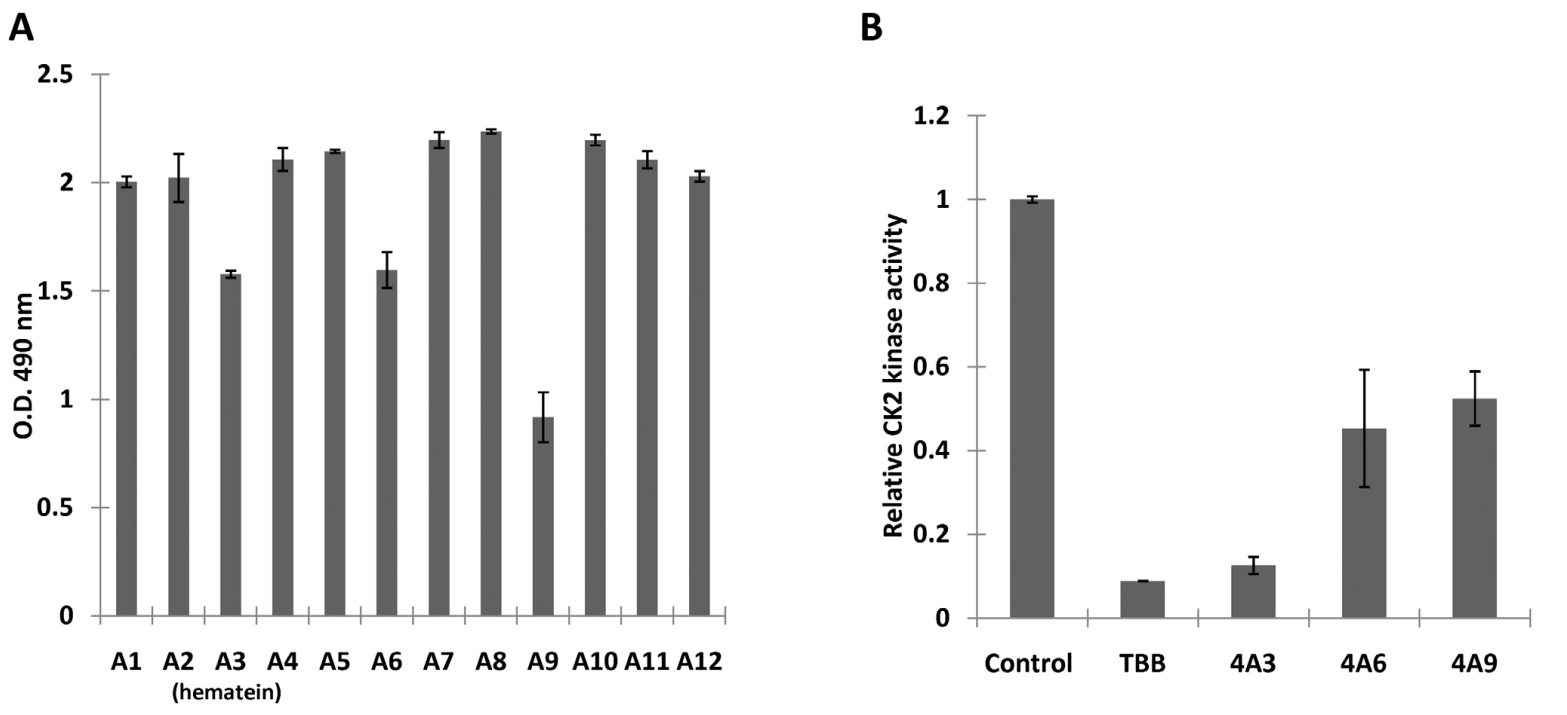
**Inhibition effects of compounds plate 4 on HCT116 cells proliferation and in-vitro inhibition effects of selected compounds on CK2 kinase activity**. A. After incubation with HCT116 cells for 48 hours, compounds 4A3 (hematein), 4A6 and 4A9 showed inhibitory effects on cell proliferation compared to the control sample (*4A1, DMSO). The longitudinal values revealed the absorbance at 490 nm recorded using an ELISA plate reader after addition of Cell Titer 96 AQueous One Solution Reagent to each well for 2 hours. Data represents the average of duplicate wells and bars indicate SD. Compound concentration: 4A3: 16.6 μM; 4A6: 23.1 μM; 4A9: 14.2 μM. B. Equal amounts (100 μM) of compounds were incubated with purified CK2, and CK2 activity was measured by Cyclex CK2 Assay/Inhibitor Screening Kit using 100 μM ATP. Ck2 kinase activity is represented as relative CK2 activity to controls (DMSO). Data represents the average of duplicate wells and bars indicate SD.4A3: hematein. Other negative results are not shown.

First, a cell based MTS cell proliferation assay was used to evaluate the inhibitory effects of 400 compounds from NPL compound library on cell proliferation of HCT116 cells. HCT116 cell was selected for initial screening because previous studies showed that this cell line was inhibited by CK2 inhibitors[17,18]. After incubation for 48 hours in 96-wells plates, a number of compounds including 4A3, 4A6 and 4A9, showed inhibition effects on HCT116 cells proliferation compared to control samples (DMSO) (Fig 1A).

Second, a non-isotopic CK2 kinase assay was performed to test the compounds selected from cell based inhibitory assay for their ability to inhibit CK2 kinase activity *in vitro*. Equal concentrations of the indicated compounds were incubated with purified CK2, and their inhibition effects were evaluated using 100 μM ATP. Compared to 4A6 and 4A9 compounds, 4A3 compound exerts stronger inhibition effect on CK2 kinase activity *in vitro *(Fig. 1B).

**Figure 2:**
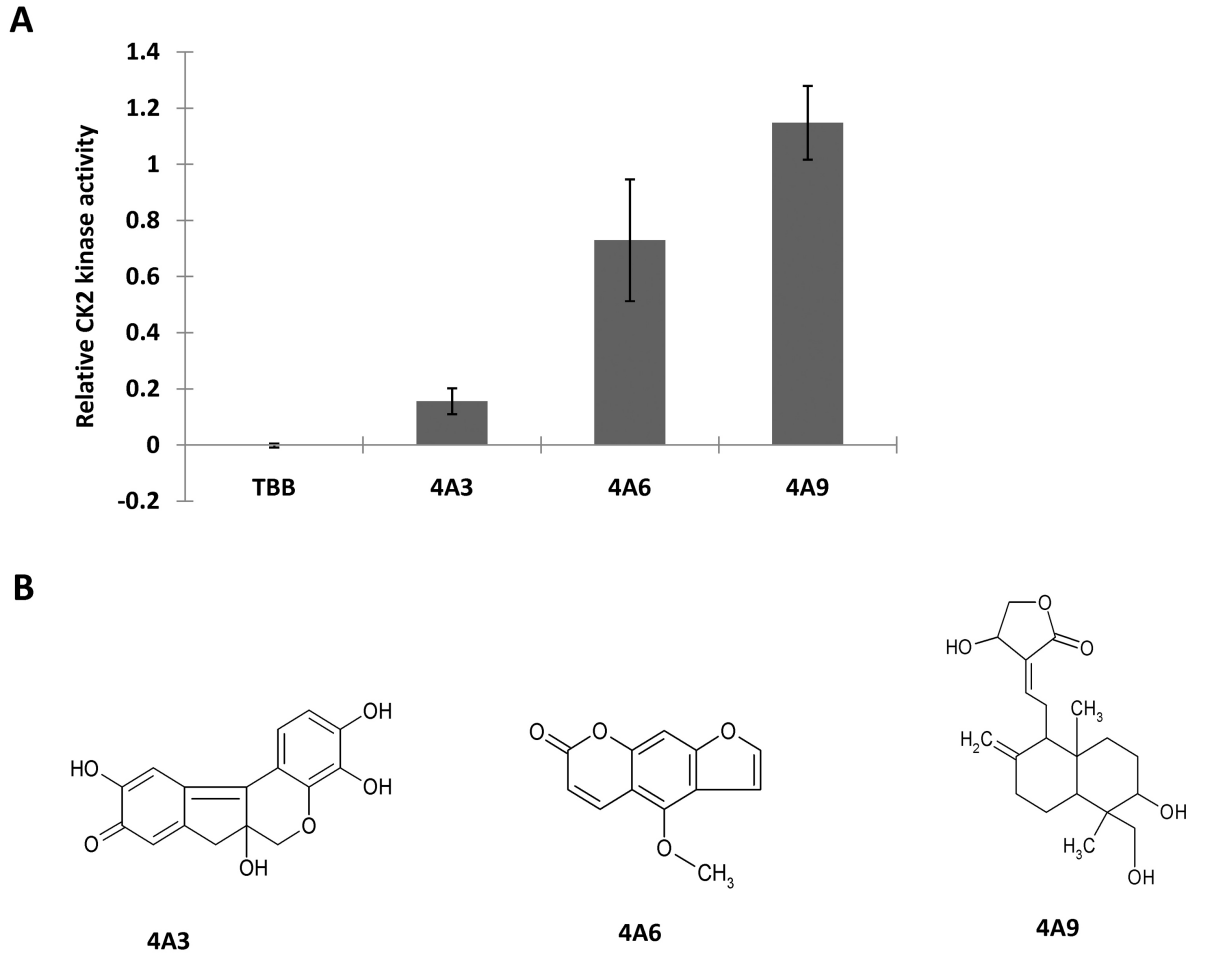
**Validation of inhibition effects of selected compounds on CK2 kinase activity and structures of compounds**. A. Inhibition effects of selected compounds on CK2 kinase activity was validated by radioisotope kinase assay at a concentration of 10 μM compound using 10 μM ATP. TBB is the positive control. Ck2 kinase activity is represented as relative CK2 activity to controls (DMSO). Data represents the average of duplicate experiments and bars indicate SD.4A3: hematein. B. Structure of compounds: 4A3 (hematein): 3, 4, 10, 6a-tetrahydroxy-7, 6a-dihydroindeno [2, 1-c] chroman-9-one MW 300.26, CAS No. 15489-90-4; 4A6: 5-methoxyfurano [3,2-g]chromen-2-one; 4A9: 4-hydroxy-3-{2- [8-hydroxy-7-(hydroxymethyl)-1,7-dimethyl-3-methylenebicyclo[4. 4.0]dec-2-yl]ethylidene}-4,5-dihydrofuran-2-one.

Third, the inhibition effects of selected compounds were further validated by radioisotope CK2 kinase assay at 10 μM ATP and similar results were noted (Fig. 2A). The 4A3 compound is hematein (3, 4, 10, 6a-tetrahydroxy-7, 6a-dihydroindeno [2, 1-c] chroman-9-one) (Fig 2B).

### Hematein inhibits CK2 kinase activity in a selective, dose-dependent and ATP non-competitive manner *in vitro*

**Figure 3:**
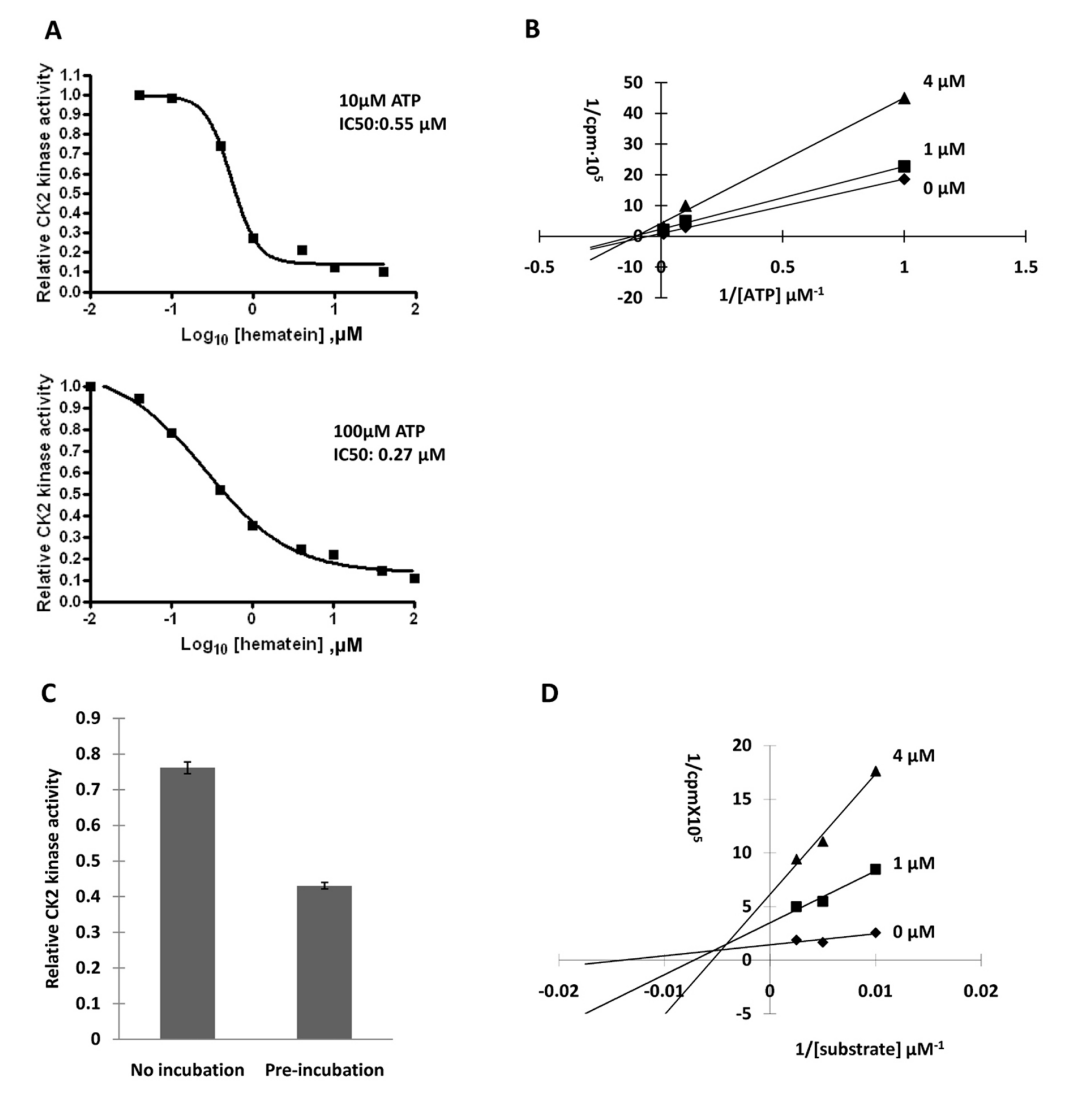
**Dose- dependent response, kinetics analysis and reversibility assay of CK2 inhibition by hematein**. A. CK2 was assayed with specific peptide described in the material and method section with the increasing concentrations of the inhibitor using 10 μM and 100 μM ATP. B. The Linewear-Burk plots illustrate the non-competitive inhibition of hematein relative to ATP toward CK2. Substrate concentration was fixed at 200 μM. CK2 activity was determined in 0 μM, 1 μ and 4 μM hematein. The data represents means of duplicate experiments with SE never exceeding 10%. C. Reversibility assay of hematein. Hematein was pre-incubated with CK2 at a concentration of 5 μM for 30 minutes and then kinase assay was performed under 0.2 μM final concentration of hematein. Ck2 kinase activity is represented as relative CK2 activity to controls (DMSO). Data points represent the average of duplicate experiments and bars indicate SD. D. The Linewear-Burk plots illustrate the mixed inhibition of hematein relative to substrate toward CK2. ATP concentration was fixed at 10 μM. CK2 activity was determined in 0 μM, 1 μM and 4 μM hematein. The data represents means of duplicate experiments with SE never exceeding 10%.

With the purpose to elucidate the specificity of hematein to CK2, a kinase panel provided by KinaseProfiler™ service (Millipore, Dundee, UK) was performed. Among 48 kinases tested in the presence of 10 μM ATP and 10 μM hematein, hematein exerts > 90% inhibition toward CK2 (Table 1). The IC_50 _value of hematein is 0.55 μM in the presence of 10 μM ATP (Fig 3A). However, the IC_50 _value did not increase in correspondence to 100 μM ATP (0.27 μM) (Fig 3A). Kinetics study was further performed and the Linewear-Burk plots showed that hematein is an ATP non-competitive inhibitor (Fig 3B).

**Table 1:** Specificity spectrum of hematein.

Protein kinase	Kinase activity (%)	Protein kinase	Kinase activity (%)
**Abl**	76	**Lyn**	111
**CDK1**	95	**MAPK1**	87
**CDK2**	113	**MAPK2**	74
**CDK3**	55	**MKK6**	99
**CDK5**	84	**MSK1**	91
**CDK7**	106	**MSK2**	72
**CHK1**	65	**mTOR**	103
**CK1**	82	**p70S6K**	61
**CK2**	**0**	**PDGFRα**	103
**cKit**	83	**PDK1**	96
**c-RAF**	102	**Pim-1**	58
**cSRC**	94	**Pim-2**	81
**DRAK1**	51	**Pim-3**	50
**DYRK2**	91	**PKA**	95
**EGFR**	31	**PKBα**	92
**Flt1**	31	**PKCα**	36
**Flt3**	83	**PKD2**	58
**GCK**	94	**PRAK**	65
**GSK3β**	147	**ROCK-II**	75
**HIPK1**	94	**SAPK2a**	121
**HIPK2**	76	**SAPK3**	99
**HIPK3**	96	**SAPK4**	112
**KDR**	62	**SGK**	92
**Lck**	113	**Syk**	136

Residual activity is determined in the presence of 10 μM hematein under 10 μM ATP and expressed as a percentage of the control without inhibitor. Residual activities ≤ 10% are shown in bold. CDK, cyclin-dependent kinase; CHK, checkpoint kinase; CK, casein kinase; SRC, sarcoma kinase; DRAK, DAP kinase-related apoptosis-inducing protein kinase; DYRK, dual-specificity tyrosine-(Y)-phosphorylation regulated kinase; EGFR, epidermal growth factor receptor; Flt, fms-related tyrosine kinase; GCK, gluokinase; GSK, glycogen synthase kinase; HIPK, homeodomain interacting protein kinase; KDR, kinase insert domain receptor; Lck, lymphocyte cell-specific protein-tyrosine kinase; MAPK, mitogen-activated protein kinase; MKK, mitogen-activated kinase; MSK, mitogen- and stress-activated protein kinase; mTOR, mammalian target of rapamyin; p70S6K, p70 S6 kinase; PDGFR, platelet-derived growth factor receptor; PDK, 3'-phosphoinositide-dependent kinase; PKA, protein kinase A; PKB, protein kinase B; PKC, protein kinase C; PKD, protein kinase D; PRAK, p38-regulated activated kinase; ROCK, Rho-dependent protein kinase; SRPK, serine-arginine protein kinase; SGK, serum and glucocorticoid-inducible kinase.

### Hematein inhibits CK2 kinase activity in a partially reversible manner *in vitro*

Reversibility test was performed with pre-incubation of hematein with CK2 in a concentration of 5 μM for 30 minutes. CK2 kinase assay was then performed with the final concentration of 0.2 μM hematein. The group with pre-incubation showed roughly 50% kinase activity of the group without pre-incubation (Fig 3C), indicating that hematein is a partially reversible inhibitor toward CK2.

### Hematein exhibits a mixed inhibition against substrate *in vitro*

The Linewear-Burk plots showed that hematein exhibited a mixed inhibition against substrate RRRDDDSDDD (Fig 3D). The apparent dissociation constants for hematein binding were calculated from secondary plots of the slopes or intercepts obtained from the Linewear-Burk plots. A secondary plot of the slopes indicated a *K*_is _value of 0.58 ± 0.09 μM (representing a reversible binding of hematein to CK2) and a secondary plot of the y-axis intercepts revealed a *K*_ii _value of 2.13 ± 0.67 μM (representing a reversible binding of hematein to CK2-substrate complex).

### Hematein inhibits CK2 kinase activity and down stream Akt phosphorylation, and induces apoptosis in A549 lung cancer cells

**Figure 4:**
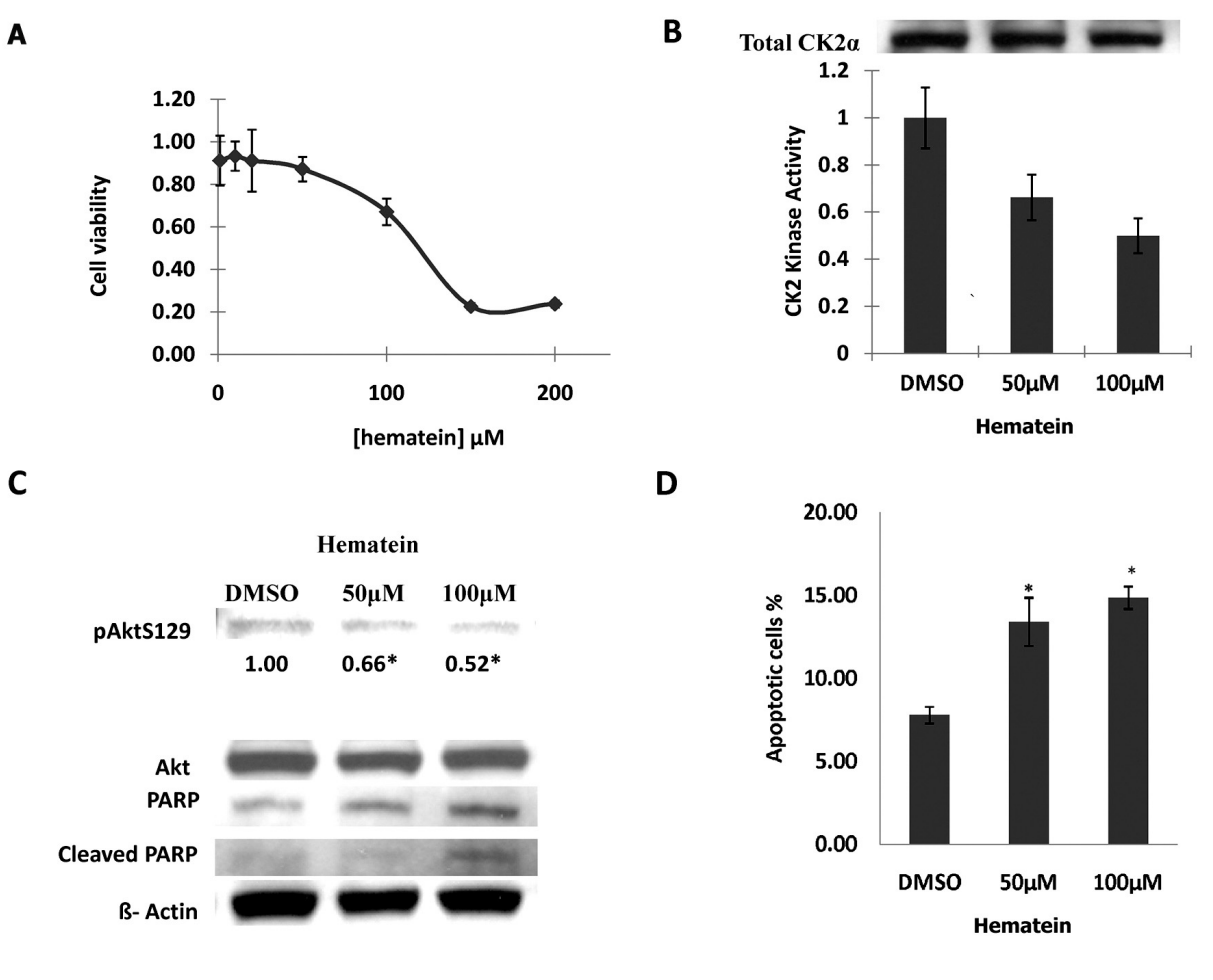
**Inhibition effects of hematein on cellular viability and kinase activity in cancer cells**. A. A549 cells were treated with serial dilutions of hematein (0 to 200 μM) and cellular viability (normalized to DMSO control) was measured after 48 hours. Data points represent the average of duplicate wells in triplet experiments and bars indicate SD. B. A549 cells were treated with DMSO (control), 50 μM and 100 μM of hematein for 48 hours. Upper western blot panel showed total amount of CK2 used for CK2 kinase assay, and lower table showed relative CK2 kinase activity (normalized to DMSO control) under different hematein concentrations. Data points represent the average of duplicate experiments and bars indicate SD. C. Phosphorylated Akt (Ser 129), total Akt, and PARP were measured by western blot analysis. β-Actin was used as internal loading control. Bands quantization of phosphorylated Akt (Ser 129) was obtained by an analysis with Quantity One 1-D analysis software. Values are reported below each band and normalized to DMSO control. "*" denotes *p *< 0.05 when compared with control values in triplet experiments. D. The fraction of cells undergoing apoptotic cell death was detected using annexin V FITC and PI stain. Data points represent the average of triplet independent experiments and bars indicate SD. "*" denotes *p *< 0.05 when compared with the control values.

Since CK2 kinase showed dose dependent response to hematein inhibition *in vitro*, we further evaluated inhibition effects of hematein on intact cancer cells. First, A549 lung cancer cells were treated with different concentrations of hematein (0 μM to 200 μM), and cellular viability was measured after 48 hours. Dose dependent response to inhibition of hematein was noted in A549 cells (Fig 4A). We next measured CK2 kinase activity in the lysate of these cells with the same amount of total protein via a radioisotope CK2 kinase assay described in materials and methods section. Dose dependent inhibition responses of CK2 kinase activity were noted in cells treated with 50 μM and 100 μM of hematein (Fig 4B). Interestingly, Akt Ser129, which is phosphorylated by CK2 *in vitro *and *in vivo*, also showed significantly decreased phosphorylation in the cells above (Fig 4C). However, total CK2, total Akt and β-actin were comparable. Increased cleaved PARP were also detected in cell lysate treated with 50 μM and 100 μM of hematein (Fig 4C), which indicated increased caspase dependent apoptosis of cancer cells after hematein treatment. Compared to DMSO treated cells, significantly increased apoptotic cells were noted in cells treated with 50 μM and 100 μM of hematein for 48 hours (Fig 4D).

### Hematein has more inhibition effects to cancer cells growth

**Figure 5:**
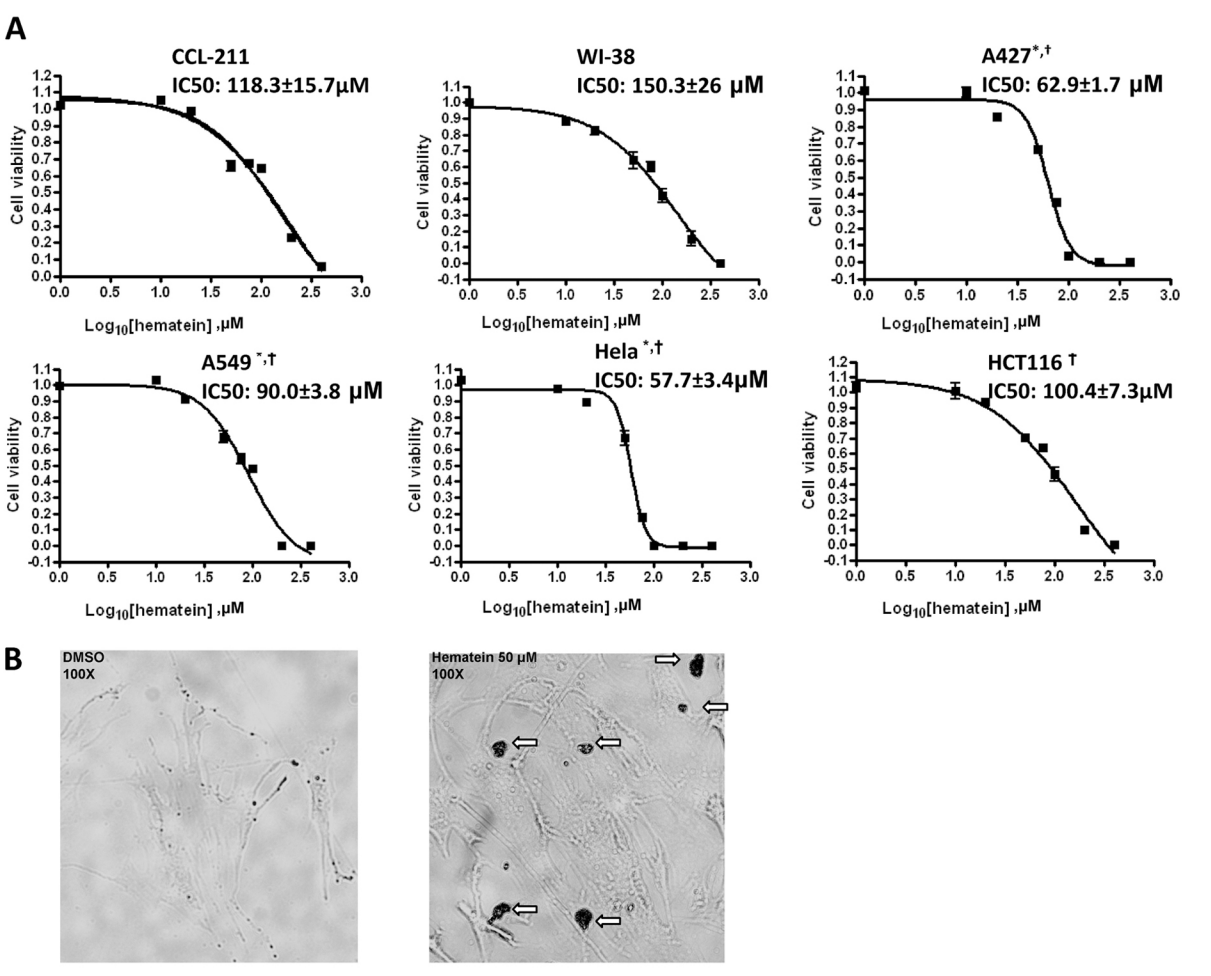
**Inhibition effects of hematein on cellular growth in normal and cancer cells**. A. Normal (WI-38, CCL-211) and cancer (Hela, A549 and A427, HCT116) cells were cultured in the absence and in increasing concentrations of hematein (10 μM to 100 μM) as indicated. Cellular viability (normalized to DMSO control) was measured after 48 hours using CellTiter-Glo^® ^Luminescent cell viability assay. Data points represent the average of IC_50 _value of hematein in triplet experiments and bars indicate SD. "*" denotes *p *< 0.05 when compared with IC_50 _values of CCL-211 cells and "†" denotes *p *< 0.05 when compared with IC_50 _values of WI-38 cells. B. CCL-211 cells were incubated in the absence and presence of 50 μM hematein for 48 hours. Block arrows indicate staining of the nucleus after hematein incubation. Original magnification: × 100.

Finally, we compared inhibition effects of hematein on normal and cancer cells. Cytotoxicity of hematein against normal and cancer cells was measured using the CellTiter-Glo luminescent cell viability assay (Promega, Madison, WI) to overcome the interference of hematein to MTS assay. Indicated cells were treated with different concentrations of hematein for 48 hours, and cell viability was measured by the CellTiter-Glo luminescent cell viability assay. From dose response curve, IC_50 _values were calculated in CCL-211 (118.3 ± 15.7 μM) and WI-38 (150.3 ± 26 μM) normal cells than in Hela (57.7 ± 3.4 μM), A549 (90.0 ± 3.8 μM), A427 (62.9 ± 1.7 μM) and HCT116 (100.4 ± 7.3 μM) cancer cells (Fig 5A). Significantly higher IC_50 _values (p < 0.05) were noted in CCL-211 cells compared to Hela, A549 and A427 cells, and in WI-38 cells compared to Hela, A549, A427 and HCT116 cells. As the result, hematein has stronger inhibition effects towards cancer cells. We also took the advantage of the staining property of hematein to observe whether normal cells uptake hematein and hematein nuclear staining was noted in cells after treated with 50 μM hematein for 48 hours (Fig 5B).

## Discussion

Cell-based screening of compound libraries have been adopted to discover possible anti-cancer small molecules in myeloma, breast cancer and colon cancer cells [19-21]. In this study, we also combined a cell based assay with kinase assay for screening CK2 inhibitors from a natural compound library. Through these assays, we identified and validated hematein as a novel CK2 kinase inhibitor.

Hematein is a natural compound isolated from *Caesalpinia sappan*, and has been used as a herbal drug in oriental medicine as both an analgesic and an anti-inflammatory agent[16] and also used in staining [22]. Our study for the first time discovered that hematein as a CK2 kinase inhibitor both *in vitro *and in cancer cells. The *in vitro *IC_50 _value of hematein (0.55 μM) on CK2 kinase activity is comparable to other CK2 inhibitors ever discovered, such as [5-oxo-5, 6-dihydroindolo-(1, 2-a) quinazolin-7-yl] acetic acid (IQA) (0.39 μM), TBB (0.50 μM) [1], 1, 3, 8-trihydroxyanthraquinone (emodin) (0.89 μM) and 2-Dimethylamino-4,5,6,7-tetrabromo-1 *H*-benzimidazole (DMAT) (0.15 μM)[15]. Kinetic assays in our study also showed that hematein is an ATP non-competitive inhibitor toward CK2. Non-competitive inhibitor of ATP has been shown to be less toxic, and has the advantage of no need to compete with high intracellular ATP concentration. Our data implies that hematein may inhibit CK2 through an allosteric mechanism. Recently, Raaf *et al *reported an allosteric binding site for the small molecule CK2 inhibitor 5,6-dichloro-1-beta-D-ribofuranosylbenzimidazole (DRB)[23]. More studies are needed to understand thoroughly the nature and mechanisms of the CK2 inhibition by hematein.

We showed that hematein also induced apoptosis of cancer cells. Earlier studies indicate that CK2 plays a key role in suppression of apoptosis. Overexpression of CK2 in cancer cells protects cells from etoposide- and diethylstilbestrol-induced apoptosis[24], results in suppression of apoptosis mediated by tumor necrotic factoralpha (TNF-α), TRAIL and Fas L, and augments apoptosis in cells responsive to these ligands[16]. It has been noted that treatment of a variety of cancer cells with cell permeable CK2 inhibitors such as TBB, IQA and DMAT results in activation of caspases and then aopotosis[1,14,25]. Hematein is reported to reduce the TNF-α-induced expression of vascular cell adhesion molecule-1 (VCAM-1) and monocyte chemotactic protein-1 (MCP-1)[16,26], and to decrease reactive oxygen species generation and nuclear factor-kappa B (NF-κB) activation [27]. In our study, hematein inhibited Akt/PKB Ser129 phosphorylation in cancer cells. Akt/PKB Ser129 plays a role in constitutive activation of Akt/PKB pathway by CK2[4], which promotes cell survival through activation of anti-apoptotic pathways such as NF-κB pathway and suppression of caspases activity[28]. Thus, hematein induces apoptosis in cancer cells at least partially through inhibition of Akt/PKB pathway by down regulation of CK2 kinase and then decreased phosphorylation of Akt/PKB Ser129.

Compared to TBB, hematein has similar on CK2 kinase inhibition *in vitro *and in cancer cells. However, TBB exerts inhibition effects on both normal and cancer cells[29]. In the contrary, hematein exerts relatively stronger inhibition effects on cancer cells than in normal cells in our study. Since hematein exerts different inhibition effects on the growth of cancer and normal cells, which is probably due to its ATP non-competitive inhibition mechanism and high selectivity. Thus, hematein may provide a clue for design of new class of CK2 inhibitors.

## Conclusion

In this study, we identified hematein as a novel CK2 small molecule inhibitor from a natural compound library and showed that hematein is a selective and cell permeable CK2 inhibitor. Hematein showed stronger growth inhibition effects to cancer cells when compared to normal cells. This compound may represent a promising class of CK2 inhibitors.

## Competing interests

The authors declare that they have no competing interests.

## Authors' contributions

MSH carried out the kinase assay, western blot analysis, and apoptosis studies and also drafted the manuscript. LY and DMJ designed the study and revised the manuscript. ZX, and YCL carried the kinase assay and cell lines experiments. JHM, CTY and PJC revised the manuscript. All authors read and approved the final manuscript.

## Pre-publication history

The pre-publication history for this paper can be accessed here:

http://www.biomedcentral.com/1471-2407/9/135/prepub

## References

[B1] Sarno S Pinna LA Protein kinase CK2 as a druggable target Mol Biosyst 2008 4 9 889 894 10.1039/b805534c 18704226

[B2] Meggio F Pinna LA One-thousand-and-one substrates of protein kinase CK2? Faseb J 2003 17 3 349 368 10.1096/fj.02-0473rev 12631575

[B3] Tapia JC Torres VA Rodriguez DA Leyton L Quest AF Casein kinase 2 (CK2) increases survivin expression via enhanced beta-catenin-T cell factor/lymphoid enhancer binding factor-dependent transcription Proc Natl Acad Sci USA 2006 103 41 15079 15084 10.1073/pnas.0606845103 17005722 PMC1622780

[B4] Di Maira G Salvi M Arrigoni G Marin O Sarno S Brustolon F Pinna LA Ruzzene M Protein kinase CK2 phosphorylates and upregulates Akt/PKB Cell Death Differ 2005 12 6 668 677 10.1038/sj.cdd.4401604 15818404

[B5] Tawfic S Yu S Wang H Faust R Davis A Ahmed K Protein kinase CK2 signal in neoplasia Histol Histopathol 2001 16 2 573 582 11332713 10.14670/HH-16.573

[B6] Seldin DC Leder P Casein kinase II alpha transgene-induced murine lymphoma: relation to theileriosis in cattle Science 1995 267 5199 894 897 10.1126/science.7846532 7846532

[B7] Landesman-Bollag E Song DH Romieu-Mourez R Sussman DJ Cardiff RD Sonenshein GE Seldin DC Protein kinase CK2: signaling and tumorigenesis in the mammary gland Mol Cell Biochem 2001 227 1–2 153 165 10.1023/A:1013108822847 11827167

[B8] Orlandini M Semplici F Ferruzzi R Meggio F Pinna LA Oliviero S Protein kinase CK2alpha' is induced by serum as a delayed early gene and cooperates with Ha-ras in fibroblast transformation J Biol Chem 1998 273 33 21291 21297 10.1074/jbc.273.33.21291 9694889

[B9] Kim JS Eom JI Cheong JW Choi AJ Lee JK Yang WI Min YH Protein kinase CK2alpha as an unfavorable prognostic marker and novel therapeutic target in acute myeloid leukemia Clin Cancer Res 2007 13 3 1019 1028 10.1158/1078-0432.CCR-06-1602 17289898

[B10] Laramas M Pasquier D Filhol O Ringeisen F Descotes JL Cochet C Nuclear localization of protein kinase CK2 catalytic subunit (CK2alpha) is associated with poor prognostic factors in human prostate cancer Eur J Cancer 2007 43 5 928 934 10.1016/j.ejca.2006.11.021 17267203

[B11] P Oc Rusch V Talbot SG Sarkaria I Viale A Socci N Ngai I Rao P Singh B Casein kinase II alpha subunit and C1-inhibitor are independent predictors of outcome in patients with squamous cell carcinoma of the lung Clin Cancer Res 2004 10 17 5792 5803 10.1158/1078-0432.CCR-03-0317 15355908

[B12] Gapany M Faust RA Tawfic S Davis A Adams GL Ahmed K Association of elevated protein kinase CK2 activity with aggressive behavior of squamous cell carcinoma of the head and neck Mol Med 1995 1 6 659 666 8529132 PMC2229971

[B13] Stalter G Siemer S Becht E Ziegler M Remberger K Issinger OG Asymmetric expression of protein kinase CK2 subunits in human kidney tumors Biochem Biophys Res Commun 1994 202 1 141 147 10.1006/bbrc.1994.1904 8037705

[B14] Ruzzene M Penzo D Pinna LA Protein kinase CK2 inhibitor 4,5,6,7-tetrabromobenzotriazole (TBB) induces apoptosis and caspase-dependent degradation of haematopoietic lineage cell-specific protein 1 (HS1) in Jurkat cells Biochem J 2002 364 Pt 1 41 47 11988074 10.1042/bj3640041 PMC1222543

[B15] Pagano MA Meggio F Ruzzene M Andrzejewska M Kazimierczuk Z Pinna LA 2-Dimethylamino-4,5,6,7-tetrabromo-1H-benzimidazole: a novel powerful and selective inhibitor of protein kinase CK2 Biochem Biophys Res Commun 2004 321 4 1040 1044 10.1016/j.bbrc.2004.07.067 15358133

[B16] Ahmad KA Harris NH Johnson AD Lindvall HC Wang G Ahmed K Protein kinase CK2 modulates apoptosis induced by resveratrol and epigallocatechin-3-gallate in prostate cancer cells Mol Cancer Ther 2007 6 3 1006 1012 10.1158/1535-7163.MCT-06-0491 17363494

[B17] Sayed M Pelech S Wong C Marotta A Salh B Protein kinase CK2 is involved in G2 arrest and apoptosis following spindle damage in epithelial cells Oncogene 2001 20 48 6994 7005 10.1038/sj.onc.1204894 11704824

[B18] Farah M Parhar K Moussavi M Eivemark S Salh B 5,6-Dichloro-ichlororibifuranosylbenzimidazole- and apigenin-induced sensitization of colon cancer cells to TNF-alpha-mediated apoptosis Am J Physiol Gastrointest Liver Physiol 2003 285 5 G919 928 12842827 10.1152/ajpgi.00205.2003

[B19] Rickardson L Fryknas M Haglund C Lovborg H Nygren P Gustafsson MG Isaksson A Larsson R Screening of an annotated compound library for drug activity in a resistant myeloma cell line Cancer Chemother Pharmacol 2006 58 6 749 758 10.1007/s00280-006-0216-7 16528529

[B20] Evans MJ Saghatelian A Sorensen EJ Cravatt BF Target discovery in small-molecule cell-based screens by in situ proteome reactivity profiling Nat Biotechnol 2005 23 10 1303 1307 10.1038/nbt1149 16200062

[B21] Teraishi F Wu S Zhang L Guo W Davis JJ Dong F Fang B Identification of a novel synthetic thiazolidin compound capable of inducing c-Jun NH2-terminal kinase-dependent apoptosis in human colon cancer cells Cancer Res 2005 65 14 6380 6387 10.1158/0008-5472.CAN-05-0575 16024641 PMC1592468

[B22] Bettinger C Zimmermann HW New investigations on hematoxylin, hematein, and hematein-aluminium complexes. II. Hematein-aluminium complexes and hemalum staining Histochemistry 1991 96 3 215 228 10.1007/BF00271540 1717413

[B23] Raaf J Brunstein E Issinger OG Niefind K The CK2 alpha/CK2 beta interface of human protein kinase CK2 harbors a binding pocket for small molecules Chem Biol 2008 15 2 111 117 10.1016/j.chembiol.2007.12.012 18291315

[B24] Guo C Yu S Davis AT Wang H Green JE Ahmed K A potential role of nuclear matrix-associated protein kinase CK2 in protection against drug-induced apoptosis in cancer cells J Biol Chem 2001 276 8 5992 5999 10.1074/jbc.M004862200 11069898

[B25] Yde CW Frogne T Lykkesfeldt AE Fichtner I Issinger OG Stenvang J Induction of cell death in antiestrogen resistant human breast cancer cells by the protein kinase CK2 inhibitor DMAT Cancer Lett 2007 256 2 229 237 10.1016/j.canlet.2007.06.010 17629615

[B26] Hong JJ Jeong TS Choi JH Park JH Lee KY Seo YJ Oh SR Oh GT Hematein inhibits tumor necrotic factor-alpha-induced vascular cell adhesion molecule-1 and NF-kappaB-dependent gene expression in human vascular endothelial cells Biochem Biophys Res Commun 2001 281 5 1127 1133 10.1006/bbrc.2001.4480 11243852

[B27] Choi JH Jeong TS Kim DY Kim YM Na HJ Nam KH Lee SB Kim HC Oh SR Choi YK Hematein inhibits atherosclerosis by inhibition of reactive oxygen generation and NF-kappaB-dependent inflammatory mediators in hyperlipidemic mice J Cardiovasc Pharmacol 2003 42 2 287 295 10.1097/00005344-200308000-00019 12883334

[B28] Duncan JS Litchfield DW Too much of a good thing: the role of protein kinase CK2 in tumorigenesis and prospects for therapeutic inhibition of CK2 Biochim Biophys Acta 2008 1784 1 33 47 17931986 10.1016/j.bbapap.2007.08.017

[B29] Ahmad KA Wang G Slaton J Unger G Ahmed K Targeting CK2 for cancer therapy Anticancer Drugs 2005 16 10 1037 1043 10.1097/00001813-200511000-00001 16222144

